# Late Miocene *Pseudolarix amabilis* bract-scale complex from Zhejiang, East China

**DOI:** 10.1371/journal.pone.0180979

**Published:** 2017-07-07

**Authors:** Yunjun Bai, Xiaoqiang Li

**Affiliations:** Key Laboratory of Vertebrate Evolution and Human Origin of Chinese Academy of Sciences, Institute of Vertebrate Paleontology and Paleoanthropology, Chinese Academy of Sciences, Beijing, China; Institute of Botany, CHINA

## Abstract

Previously, the identification of fossil *Pseudolarix* at the species level has been based on the morphology of the bract-scale complex of the seed cone. The morphological consistence of fossils through most of the Cenozoic with extant *P*. *amabilis* has led them to be considered conspecific, suggesting that *P*. *amabilis* is an extraordinary example of morphological stasis. However, the lack of cuticular evidence, especially for the leaf-homologous bract, reduces the accuracy of fossil identification based on morphology, thus weakening the evidence for morphological stasis in *P*. *amabilis*. For the first time, we provide cuticular evidence of the bract-scale of fossil *P*. *amabilis* based on the bract-scale complex from the late Miocene Shengxian Formation, Zhejiang, East China, which improves the identification accuracy and reinforces the concept of morphological stasis in this species. Second, we preliminarily reveal the niche stability of *P*. *amabilis*, which corresponds to its morphological stasis. Finally, we infer that the late Miocene forest containing *P*. *amabilis* in Zhejiang was an evergreen sclerophyllous broad-leaved or mixed mesophytic forest, which combined with the evergreen broad-leaved forest suggested by previous megafossil studies, indicates the occurrence of vertical vegetation zonation.

## Introduction

Today, *Pseudolarix* Gordon (Pinaceae) is a monotypic genus represented by the single species *P*. *amabilis* (Nelson) Rehder [1]. The unique biological features of *Pseudolarix* that distinguish it from other members of the Pinaceae are its combination of deciduous bract-scale complexes and needles and branch dimorphism. The dimorphic branching system is characterized by needles borne helically in long shoots (leading shoots) and fascicularly in short shoots (brachioblasts) [1].

**Fig 1:**
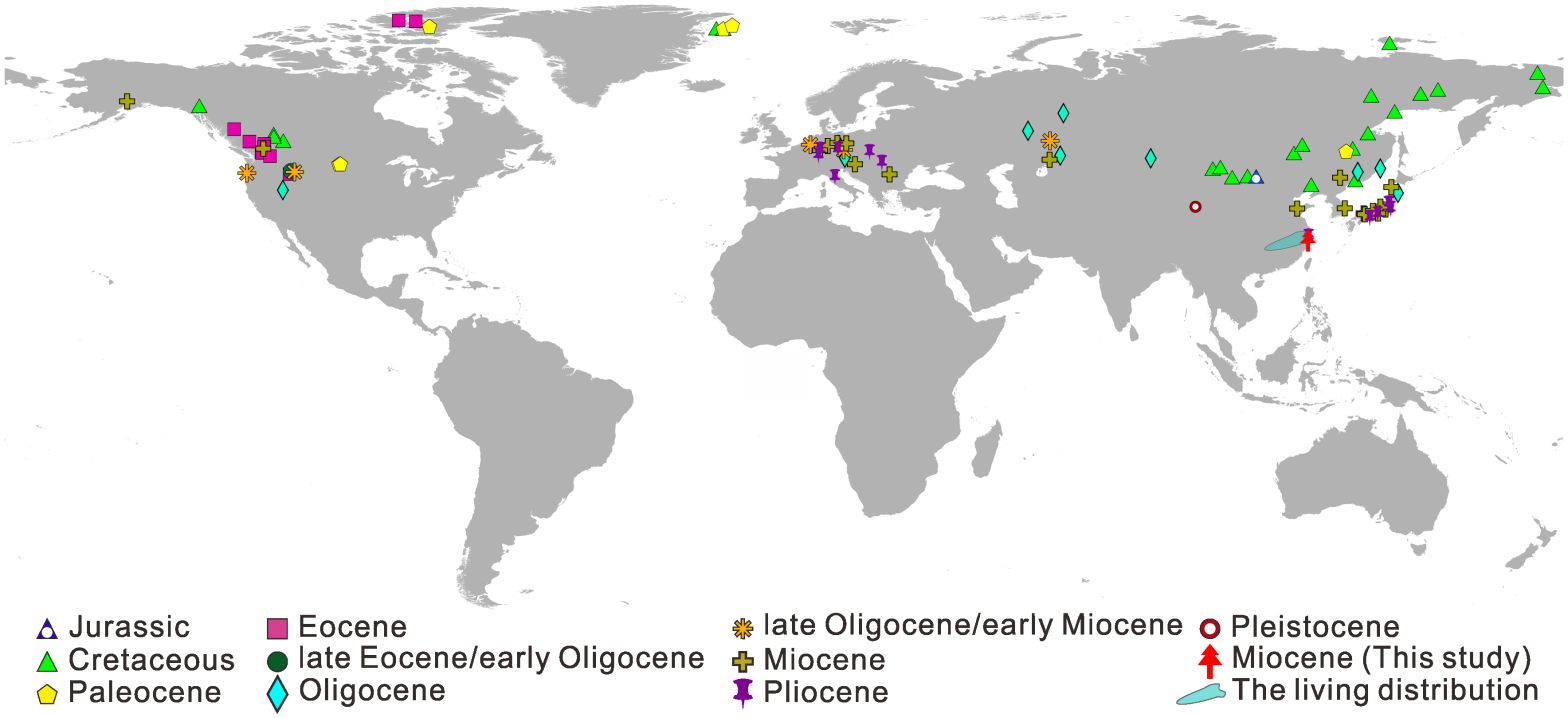
The worldwide distribution of fossil and living *Pseudolarix*. Fossil records are updated based on LePage and Basinger [5]; for updated fossil records, see S1 Table. Living distribution data are accessed through Chinese Virtual Herbarium (CVH) Data Portal (http://www.cvh.ac.cn/) (S2 Table). The map is generated by ArcGis v. 9.3 (http://www.arcgis.com/home/index.html).

As an endemic genus to China, *Pseudolarix* is now highly restricted to the lower Yangtze River valley in Southeast China [1, 2], whereas the geographical distribution of fossil *Pseudolarix* is much wider (Fig 1). *Pseudolarix* fossils are preserved as seed cones, seeds, needles, wood, brachioblasts and pollen and have been widely reported geographically from Eurasia and North America and stratigraphically from the late Jurassic to the Pleistocene (Fig 1). The earliest *Pseudolarix* (*Pseudolarix* sp.) fossil was found in the upper Jurassic (ca. 156 Ma) Tsagaan Tsav Formation in southeastern Mongolia [3], indicating that *Pseudolarix* is the oldest known fossil record for any extant Pinaceae genus, and this finding has been confirmed by molecular phylogenetic analyses [4].

The gradual climate cooling that occurred in the Northern Hemisphere by the Miocene might be responsible for the shrinking distribution of *Pseudolarix*, and the Quaternary climate deterioration, which was marked by the well-known Quaternary glaciation, may have driven *Pseudolarix* to extinction in Europe, North America and most of Asia [6, 7]. For example, the eradication of *Pseudolarix* in Europe may have resulted from the considerable cooling and drying after the Pliocene [8] and that in Japan may be related to the drying climate during the latest Pliocene [9]. Compared to most of the Northern Hemisphere, the topography of Southeast China is highly diverse, which might have mitigated the climatic disturbances resulting from the significant cooling in the Quaternary [10]. Consequently, the relatively stable climate of Southeast China allowed this region to become an excellent biological refuge from catastrophic climate deterioration, in which *P*. *amabilis* survived as a relict species.

Although abundant *Pseudolarix* fossils have been discovered, only two species have been identified based solely on the bract/scale length ratio [5]. One was assigned to an extinct species, *P*. *wehrii* Gooch, due to long bracts that are greater than 50% of the length of the ovuliferous scales; this species has only been reported in Eocene sediments in North America [5, 11]. The other species, *P*. *amabilis*, is extant and characterized by short bracts less than 50% of the length of the ovuliferous scales. Compared with *P*. *wehrii*, the distribution of *P*. *amabilis* is wider, and fossils of this species have been reported stratigraphically from the Eocene, Oligocene, Miocene and Pliocene and geographically in North America and Eurasia [5]. The earliest fossil *P*. *amabilis* was discovered in the early Eocene Allenby Formation in British Columbia [5, 11–13], and the morphological-based classification of fossils over most of the Cenozoic to the living *P*. *amabilis* leads *P*. *amabilis* to be regarded as an extraordinary example of morphological stasis [5].

Given that bracts and ovuliferous scales are leaf and reproductive shoot homologues, respectively [14–17], cuticular evidence, especially for the bracts, is much more important to definitively identify fossil *Pseudolarix*. However, this information has been lacking until now, compromising the accurate discrimination of fossils to the level of species and challenging the determination of morphological stasis for *P*. *amabilis*, although the anatomical structure of the cross section of fossil *Pseudolarix* ovuliferous scale has been described by LePage and Basinger [5].

Here, we analyse the cuticles of fossil *P*. *amabilis* from the late Miocene Shengxian Formation, Zhejiang, East China, assess niche stability in *P*. *amabilis*, and explore its implications for the paleovegetation.

## Materials and methods

**Fig 2:**
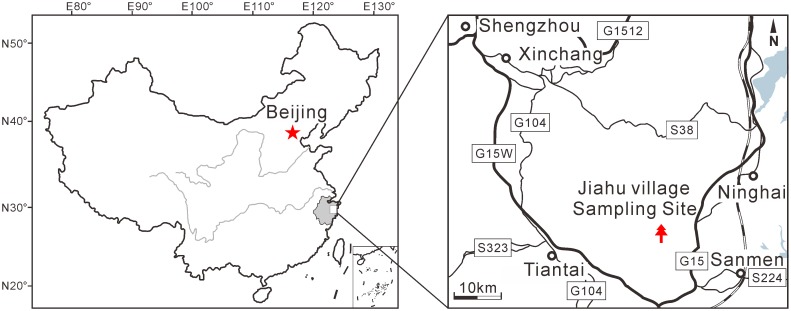
Maps showing the fossil locality at Jiahua village, Tiantai County, Zhejiang, East China. The maps are created by CorelDRAW X8 (http://www.coreldraw.com/en/product/graphic-design-software/).

The described fossil bract-scale complexes were collected from the Shengxian Formation outcrop near Jiahua village, Tiantai County, Zhejiang, East China (29.2°N, 121.2°E; Fig 2). ^40^Ar-^39^Ar dating indicates the age of the Shengxian Formation to be 10.5±0.5 Ma [18]. The herbarium sheets used for comparison were obtained from the PE Herbarium, Institute of Botany, Chinese Academy of Sciences, Beijing, China. These herbarium sheets include *P*. *amabilis* (Col. Nos. 18850, 74383, 1169, 18591, 28492), *Cedrus deodara* (Col. No. 2763), *Abies alba* (Col. No. N/A), *Araucaria cunninghamii* (Col. No. 235) and *Agathis dammara* (Col. No. 2021).

Fossil bract-scale cuticles were treated with Schulze’s solution, while extant bract-scale cuticles were treated with a mixture of 30% H_2_O_2_ and 99% CH_3_COOH (1:1 by volume). The cuticles were observed under a light microscope (Nikon Eclipse LV100POL) and an environmental scanning electron microscope (Zeiss Evo MA25). Fossil specimens are housed in the Institute of Vertebrate Paleontology and Paleoanthropology, Chinese Academy of Science, Beijing, China.

## Results

### Description of *Pseudolarix amabilis* from the late Miocene of Zhejiang

Order—Coniferales Gorozhankin, 1904

Family—Pinaceae Lindley, 1836

Genus—*Pseudolarix *Gordon, 1858

Species—*Pseudolarix amabilis* (Nelson) Rehder, 1919

**Materials**—Fossil specimens SX1, SX2 and SX3.

**Fig 3:**
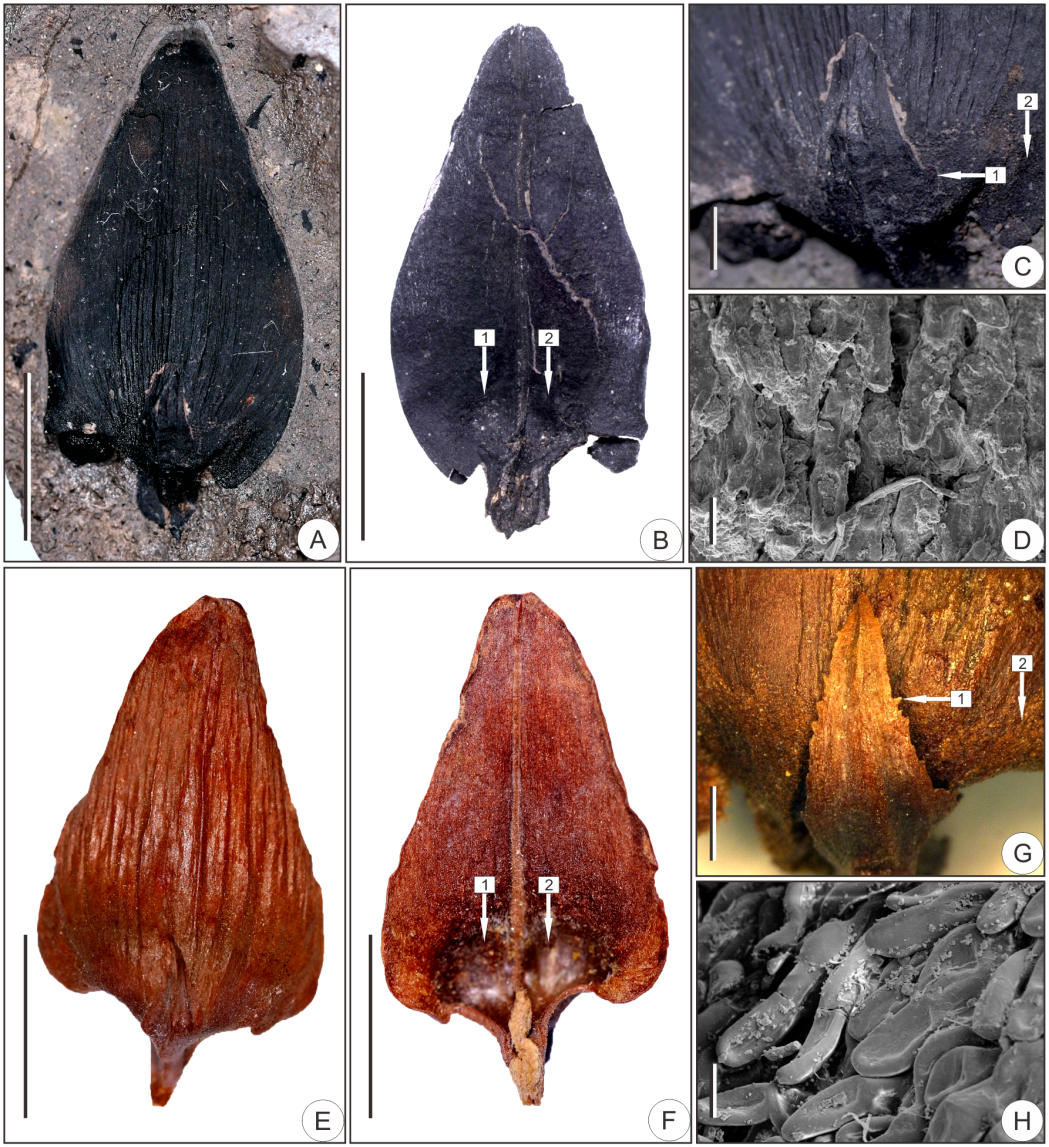
Bract-scale complex morphology of fossil and living *Pseudolarix amabilis*. (A–D) Fossil specimen SX1. (A) Abaxial surface of the bract-scale complex. (B) Adaxial surface of the ovuliferous scale with arrowheads 1 and 2 pointing to the two seed impressions. (C) Amplification of the basal part of the abaxial bract-scale complex surface showing bract serrations and the pubescence insertion position, which are indicated by arrowheads 1 and 2, respectively. (D) Pubescence morphology. (E–H) Extant species for comparison. (E) Abaxial surface of the bract-scale complex. (F) Adaxial surface of the ovuliferous scale. (G) Amplification of the basal part of the abaxial bract-scale complex surface. Arrowheads in (F) and (G) point to the corresponding parts in (B) and (C), respectively. (H) Pubescence. Scale bars: (A, B, E, F), 1 cm; (C, G), 2 mm; (D, H), 50 μm.

**Fig 4:**
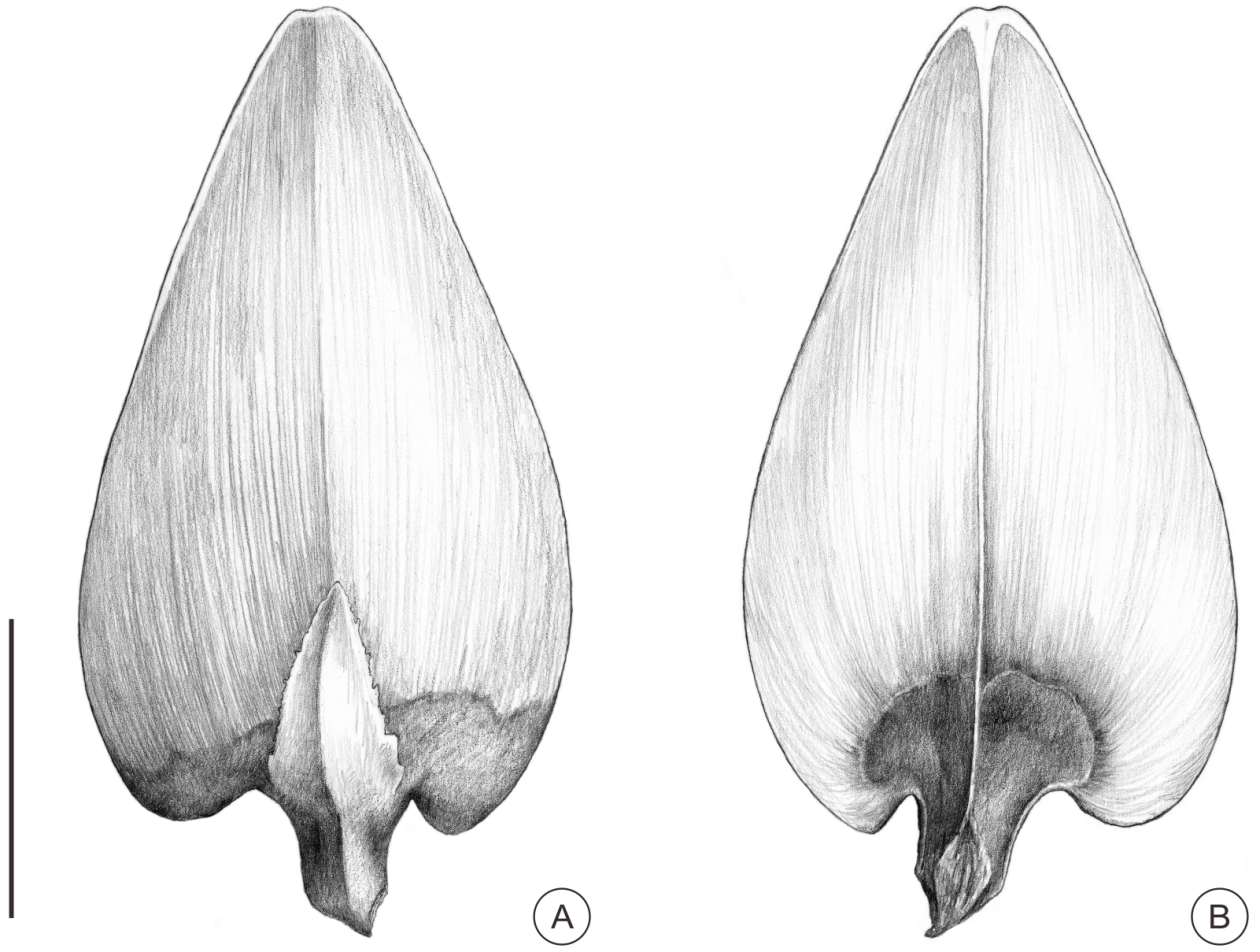
Reconstruction of fossil *Pseudolarix amabilis*. (A) Abaxial surface of the bract-scale complex. (B) Adaxial surface of the ovuliferous scale. Panels (A, B) were drawn by Aili Li. Scale bars: (A, B), 1 cm.

**Morphology**—The woody ovuliferous scales are deltoid-triangular in shape, ca. 26.9 mm long (Figs 3A, 3B, 4A and 4B) and 10.6–15.7 mm wide (Figs 3A, 3B, 4A and 4B, S1 Fig). The margins are entire (Figs 3A, 3B, 4A and 4B, S1 Fig); the apices are emarginated (Figs 3A, 3B, 4A and 4B); and the bilateral bases are auriculate (Figs 3A, 3B, 4A and 4B, S1 Fig). The widest parts are located at the lower third of the ovuliferous scales (Figs 3A, 3B, 4A and 4B, S1 Fig). There is a short pedicel, ca. 1.9–4.6 mm long at the base of each ovuliferous scale (Figs 3A, 3B, 4A and 4B, S1 Fig), and obvious longitudinal stripes occur at the abaxial surfaces (Figs 3A and 4A, S1 Fig). The basal abaxial surfaces are puberulent, while the medial and apical parts are glabrous (Figs 3C, 3D and 4A). Each pubescence is composed of single or multiple cells (Fig 3D). Two seed impressions are apparent at the bases of each glabrous adaxial surface (Figs 3B and 4B, S1 Fig).

Bracts are located at the bases of ovuliferous scales and are adnate to scales basally and detached medially and apically (Figs 3A, 3C and 4A, S1 Fig). They are ovate-lanceolate, 2.4–4.0 mm wide (Figs 3A, 3C and 4A, S1 Fig). The margins are serrated (Figs 3C and 4A, S1 Fig). 1–3 blunt serrates preserved at the base (Fig 3C, S1 Fig), while serrates at the middle and upper parts may have been lost with apparent broken traces remaining (S1 Fig). The bracts of both specimen SX1 and SX3 do not exceed 50% of the length of the ovuliferous scales due to the loss of the top apex of the bracts (Figs 3A and 4A, S1 Fig). Although the bract of specimen SX2 is missing, it is reasonable to consider the SX2 bract to be less than 50% of the length of the ovuliferous scale based on the close association of specimen SX2 with SX1 and SX3.

**Fig 5:**
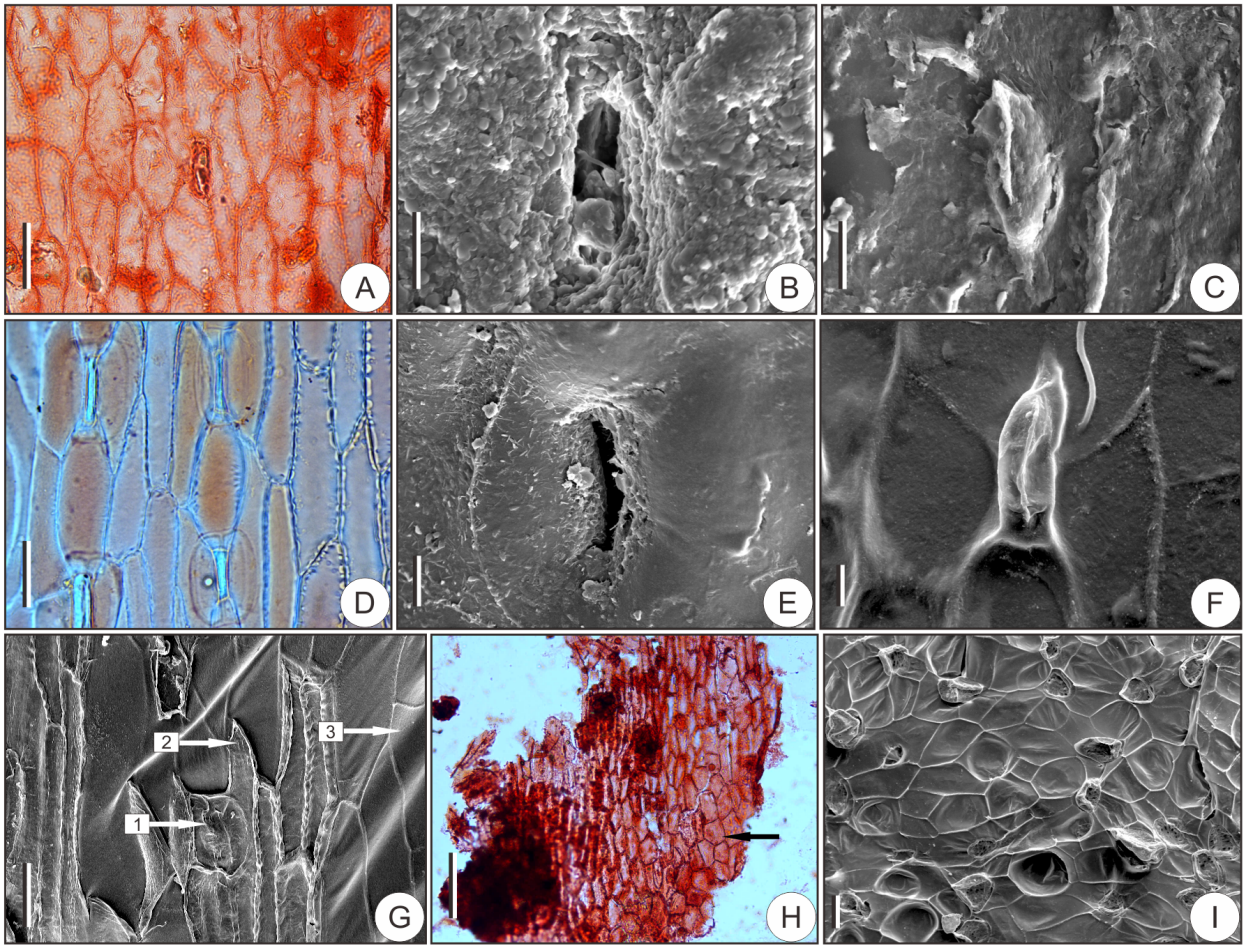
Ovuliferous scale epidermis of fossil and living *Pseudolarix amabilis*. (A–C, H) Abaxial fossil cuticle. (A) Cuticle of the upper half of the ovuliferous scale showing the arrangement of epidermal cells and stomata. (B) Outer surface of the cuticle showing the sunken stomatal apparatus. (C) Inner surface of the cuticle showing the cuticular projection resulting from the sunken stomatal apparatus. (D-G, I) Abaxial epidermis of extant species for comparison. (D) Epidermis of the upper half of the ovuliferous scale. (E) Outer surface of the sunken stomatal apparatus. (F) Cuticular projection on the inner surface of the cuticle. (G) Cuticle with epidermal cells attached with arrowhead 1 pointing to the stoma, arrowhead 2 pointing to the epidermal cell, and arrowhead 3 pointing to the cuticle. (H) Cuticle of the auriculate parts of the fossil ovuliferous scale with the approximate isodiametric polygon epidermis cells indicated by an arrowhead. (I) Cuticle at the base of the auriculate parts of the extant ovuliferous scale. Scale bars: (A, D, G, I), 40 μm; (B, C, E, F), 10 μm; (H), 100 μm.

**Cuticles**—Stomata are mainly distributed across the upper half of the abaxial surfaces of the ovuliferous scales (Fig 5A). The sunken stomatal apparatuses are longitudinally oriented (Fig 5A and 5B), and the stomatal pores open between adjacent epidermal cells (Fig 5A). Cuticular projections resulting from the sunken stomatal apparatuses appear on the inner surfaces of the abaxial cuticles (Fig 5C). The abaxial epidermal cells at the bases of the auriculate parts form approximate isodiametric polygons (Fig 5H), while at remaining parts are primarily longitudinally elongate (Fig 5A). No clear cell outlines are observed on the very thin adaxial cuticles of the ovuliferous scales.

**Fig 6:**
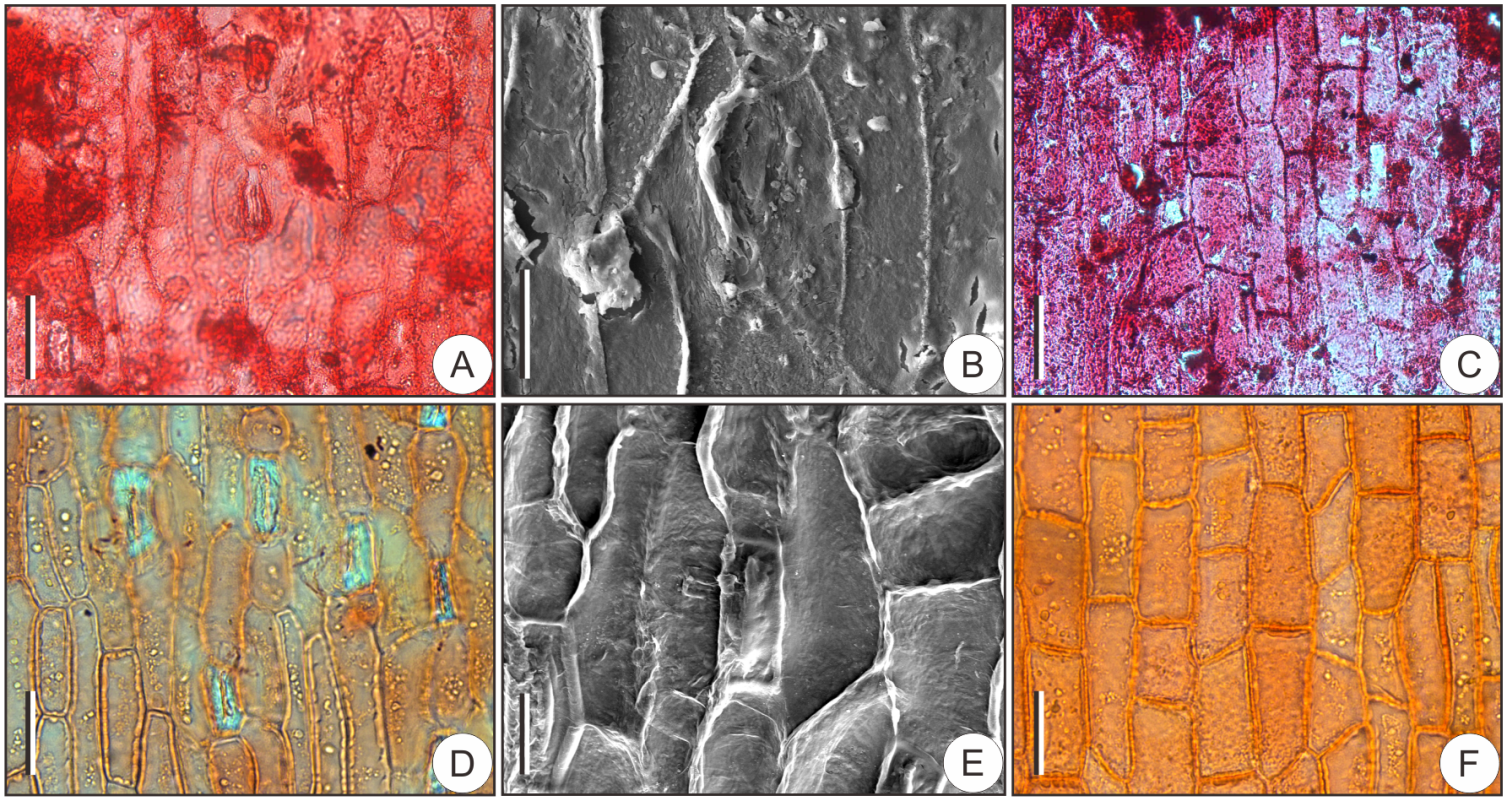
Bract epidermis of fossil and living *Pseudolarix amabilis*. (A–C) Fossil cuticle. (A) Adaxial cuticle showing the arrangement of epidermal cells and stomata. (B) Inner surface of the stomatal apparatus showing the monocyclic subsidiary cells. (C) Abaxial cuticle showing the rectangular epidermal cells. (D–F) Cuticle of extant species for comparison. (D) Adaxial epidermal cells and stomata. (E) Stomatal apparatus. (F) Abaxial epidermal cells. Scale bars: (A, C, D, F), 40 μm; (B, E), 20 μm.

Abundant stomata appear on the adaxial surfaces of bracts and are longitudinally oriented (Fig 6A). Stomatal apparatuses are superficial, i.e., not sunk beneath the adjacent epidermal cells (Fig 6A and 6B). Subsidiary cells are monocyclic (Fig 6A and 6B). Adaxial epidermal cells are longitudinally elongate (Fig 6A). No stomata appear on the abaxial epidermises, on which only rectangular epidermal cells occur (Fig 6C).

## Discussion

### Taxonomic considerations

The bract-scale complexes of conifers are persistent or deciduous after seed cone maturity [19], and the fossil bract-scale complexes studied here appear individually and integrally, which implies that the complexes are originally deciduous. Five conifer genera with deciduous bract-scale complexes are *Cedrus*, *Abies*, *Araucaria*, *Agathis* and *Pseudolarix* [19]. The scales of *Cedrus* are flabellate-obtriangular, and the bracts are minute (S2 Fig); the scales of *Abies* are reniform, trapeziform or flabellate, and the bracts are oblong, obcordate or obovate (S2 Fig); the scales of *Araucaria* are ligulate, and the bracts are woody, with reflexed or upcurved apex (S2 Fig); the fused bract-scale complexes of *Agathis* are flabellate, with thickened, recurved, nonprojecting apex (S2 Fig). Therefore, the fossil bract-scale complexes are apparently distinct from those of *Cedrus*, *Abies*, *Araucaria* and *Agathis*.

Based on previous morphological investigations [1, 5] and our first comprehensive epidermal surveys of the *Pseudolarix* bract-scale complex, we can provide a systematic description of the biological characteristics of the *P*. *amabilis* bract-scale complex.

The woody ovuliferous scales of *P*. *amabilis* are deltoid or ovate-triangular in shape (Fig 3E and 3F), 6.5–35.0 mm long and 2.5–19.0 mm wide (Fig 3E and 3F, ref. [5]). The abaxial scale surfaces are longitudinally striated with obvious midribs (Fig 3E), and the widest parts of the scales are near the lower third (Fig 3E and 3F). The margins are entire (Fig 3E and 3F), and the apices are acute, emarginate, rounded, or bluntly acute (Fig 3E and 3F, ref. [5]). The bases are typically auriculate with 2 lateral auricles (Fig 3E and 3F), and there is a short (1.0–5.0 mm) basal triangular pedicel linking each bract-scale complex and the central axis of the seed cone (Fig 3E and 3F, ref. [1]). The basal parts of the abaxial surfaces are puberulent, while the medial and apical parts are glabrous (Fig 3G and 3H). Each pubescence is composed of 1–4 cells (Fig 3H). The adaxial surfaces are glabrous, and two seeds are located at the bases of these surfaces (Fig 3F).

The bracts subtending the ovuliferous scales of *P*. *amabilis* are ovate-lanceolate in shape (Fig 3E and 3G), 3.4–12.0 mm long and 1.8–4.0 mm wide [5]. The mean bract/scale length ratio is 0.38:1 [5]. The margins are serrated (Fig 3G), and the apices are acute or acuminate (Fig 3G, refs. [1, 5]). The bracts are adnate to the ovuliferous scales at the base but are free acropetally (Fig 3G, ref. [1]).

The longitudinally oriented stomatal apparatuses are predominately distributed on the upper half of the abaxial surfaces of the ovuliferous scales (Fig 5D). Stomatal apparatuses are sunken (Fig 5E and 5F) and distributed under one layer of epidermal cells (Fig 5G), and the stomatal pores open between adjacent epidermal cells (Fig 5D). The cuticular projections on the inner surfaces of the abaxial cuticles appear after the epidermal cells are removed (Fig 5F), and it is only through the retained epidermal cells that the monocyclic subsidiary cells can be observed (Fig 5G, S3 Fig). This may explain why the stomata morphology cannot be observed on fossil cuticles. Two morphologically distinct types of epidermal cells occur at the abaxial epidermises, i.e., approximate isodiametric polygon-shaped cells at the base of auriform parts (Fig 5I) and longitudinally elongate cells on the remaining parts (Fig 5D). The ovuliferous scale adaxial cuticles are very thin. No stomata occur on the adaxial surfaces, and the adaxial epidermal cells are mainly rectangle in shape (S3 Fig).

In contrast to the epidermal patterns on ovuliferous scales, the epidermal structures on bracts exhibit the opposite configuration. On the adaxial epidermises of bracts, the epidermal cells are longitudinally elongate (Fig 6D); the stomata are superficial and longitudinally oriented (Fig 6D and 6E); and the subsidiary cells are monocyclic (Fig 6E). In contrast, on the abaxial epidermis, no or extremely rare (ca. 1–2) stomata occur, and the epidermal cells are mainly rectangular (Fig 6F).

Comparing the morphological and cuticular characteristics of the fossils and extant bract-scale complexes, we find the character combination of the fossils falls within the circumscription of *P*. *amabilis*. Consequently, the fossils described herein can be accurately assigned to *P*. *amabilis*.

### Comparisons with the fossil *P*. *amabilis* bract-scale complexes

LePage and Basinger [5] reviewed the fossils *Pseudolarix* and re-identified the fossils at species level based on the morphology of the bract-scale complexes in 1995. Using their identification criteria, we do not recognize any new species-level fossil after revisiting the published fossils of *Pseudolarix* since 1995.

Compared with previous studies of fossil *Pseudolarix*, this research is the first to analyse the cuticles of fossil bract-scale complexes and accurately assign them to the living *P*. *amabilis*. The agreement of *P*. *amabilis* fossil identification from cuticular evidence with that based on morphological features reinforces the concept of morphological stasis in *P*. *amabilis*.

Two impression fossils of *Pseudolarix* ovuliferous scale were once reported from the Pliocene sediments in Yuyao, Zhejiang [20], which is north of our study site. The fossils described here represent the southern-most record of *P*. *amabilis* worldwide and the earliest fossil *P*. *amabilis* in the extant distribution. This finding indicates that *P*. *amabilis* arrived in Zhejiang, East China by the late Miocene, which is significant for understanding the biogeography of *Pseudolarix*.

### Niche stability of *P*. *amabilis* and its paleoecological significance

Species adapt to changing environments by maintaining their ancestral niche parameters or altering aspects of their ecological niche [21], and the ecological tolerance of a species is primarily controlled by its morphology [22]. Consequently, morphological stasis is theoretically related to niche stability, while morphological change is associated with niche evolution [23–25]. Therefore, as a typical morphologically static species, *P*. *amabilis* should be characterized by niche stability over time, but this remains unexamined.

Ecological niche modelling (ENM) is considered to be an effective tool for evaluating the niche stability of a species over time [25]. The fundamental principle underlying ENM assessment is the qualitative and quantitative analysis of the degree of geographical overlap when the niche model for one time slice is projected onto the environmental layers of the second time slice [25–28]. Rich fossil records with statistical significance are a prerequisite for accurate modelling, but *P*. *amabilis* fossil records during certain time intervals are rare. For example, the richest fossil records of *P*. *amabilis* at a certain time slice in the world are represented by records from six early Pliocene fossil sites in Japan [5], so the scarcity of *P*. *amabilis* fossils hinders the application of ENM. In this case, direct comparisons of the ecological requirements of *P*. *amabilis* during different periods since the earliest reported fossil *P*. *amabilis* represent an alternative method for assessing the niche stability of *P*. *amabilis*.

**Fig 7:**
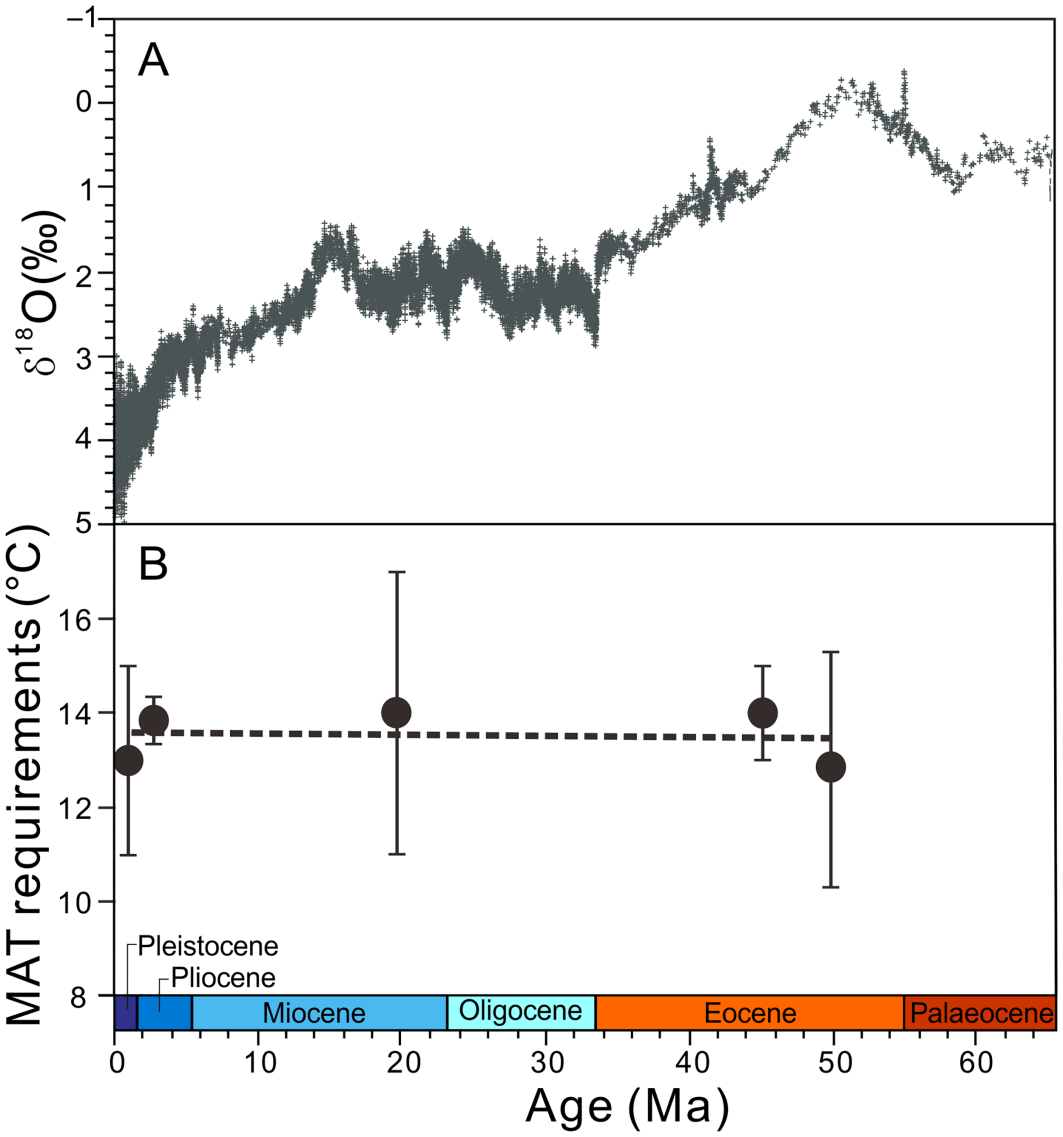
MAT requirements for the survival of *Pseudolarix amabilis* compared with global climate over the Cenozoic. (A) Global benthic δ^18^O record (modified from Zachos et al. [7]). (B) MAT requirements of *P*. *amabilis* at different time slices during the Cenozoic (for details, see Table 1). The dashed line shows the linear trend in the median required MAT values.

Temperature conditions are important components of the ecological niche of a species [29]. Accordingly, in the context of the substantial cooling in the Cenozoic [6, 7], the mean annual temperature (MAT) requirements for the survival of *P*. *amabilis* under different climatic conditions can partly reflect its niche stability. Fossils of *P*. *amabilis* have been reported from time slices of the early Eocene, middle Eocene, early Miocene and late Pliocene [5], and these time intervals, combined with data from the present, can represent the primary stages of Cenozoic temperature changes (Fig 7A). Here, we use the MAT estimates for the above five time slices at different localities worldwide to represent the MAT requirements of *P*. *amabilis* under different ecological environments. The relative consistency among the MATs suggests that no obvious changes occurred in the MAT requirements of *P*. *amabilis* (Fig 7B, Table 1), further indicating its niche stability to a certain degree.

**Table 1:** The MAT requirements of *Pseudolarix amabilis* through geological time^a^.

Age	MAT (°C)	Methods	Locations	References
Early Eocene	10.3–15.3	BA^b^	Allenby Formation: Allison Creek and Coalmont Tulameen Road, British Columbia	LePage and Basinger [5], Gooch [11], Dillhoff et al. [12], Hills and Baadsgaard [13]
Middle Eocene	13–15	LMA^c^, Isotopic Analysis	Buchanan lake Formation: Axel Heiberg Island, Canadian High Arctic	LePage and Basinger [5], Greenwood et al [30], Jahren and Sternberg [31]
Early Miocene	11–17	LMA^c^	Bitterfeld floral complex: Muldenstein, Saxony, Germany	Mai [32], Mai and Walther [33], Mai and Walther [34], Roth-Nebelsick et al. [35]
Late Pliocene	13.8–14.4	CA^d^	Reuver Formation: Reuver, Northwest Germany	Reid and Reid [36], Laurent and Marty [37], Utescher et al. [38], Florschütz [39]
Current	11–15	Meteorological station data	Lower Yangtze River valley, Southeast China	Wang [40], Zanni and Ravazzi [41], Wolfe [42], Wolfe [43]

^a^The fossil taxa used in BA, LMA and CA for paleo-MAT reconstructions do not include *Pseudolarix*, which avoids a circular argument in the estimates of the MAT requirements of *P*. *amabilis*.

^b^BA: bioclimatic analysis.

^c^LMA: leaf-margin analysis.

^d^CA: coexistence approach.

The ecological niche of a species primarily controls its geographic distribution [25]. The set of environmental conditions of a geographic region constrain the types of vegetation that develop, and the vegetation of a geographic region can reflect the local environment. As the flora in which *P*. *amabilis* naturally survives provides some indication of its ecological niche, examining the flora containing *P*. *amabilis* over geological time can elucidate its niche stability in part. The representative flora containing *P*. *amabilis* includes the early Eocene Thomas Ranch flora [12], the middle Eocene Axel Heiberg flora [30, 31], the early Miocene Bitterfeld flora [32–35], the late Pliocene Reuver flora [36, 37] and the current forest of the lower Yangtze River valley of Southeast China [40, 41]. These floras suggest a dominant evergreen sclerophyllous broad-leaved or mixed mesophytic forest type. Consequently, the consistency among flora types during different periods over the Cenozoic also suggests the niche stability in *P*. *amabilis* to some extent.

Under niche stability, plants shift their distribution in response to climate change as species move into newly favourable territories and retreat from increasingly hostile areas [44–46]. Therefore, the extinction of *P*. *amabilis* throughout most of the world over geological time and its resultant, current restriction in Southeast China may be attributed to the niche stability of this species during significant Cenozoic climate change.

Niche stability in *P*. *amabilis* is significant for reconstructing the late Miocene vegetation of Zhejiang, East China because it indicates an evergreen sclerophyllous broad-leaved or mixed mesophytic forest, while previous studies of megafossil flora have suggested an evergreen broad-leaved forest [20, 47]. Vertical vegetation zonation is presumed to reconcile the coexistence of different kinds of forests, with evergreen broad-leaved forests primarily dominated by Fagaceae at lower altitudes and evergreen sclerophyllous broad-leaved or mixed mesophytic forests, including *P*. *amabilis*, at higher altitudes. The late Miocene vegetation scenario is similar to that of today, i.e., characterized by evergreen broad-leaved forests growing below an altitude of 800 m and evergreen and deciduous broad-leaved mixed forests and deciduous forests above 800 m [48].

## Supporting information

S1 Table The updated fossil *Pseudolarix amabilis*. (DOCX)

S2 Table The occurrence of living *Pseudolarix amabilis* in China. (DOCX)

S1 Fig Fossil *Pseudolarix amabilis*. (A, B) Ovuliferous scale of specimen SX2. (A) Abaxial surface. (B) Adaxial surface with arrowheads 1 and 2 pointing to the two seed impressions. (C) Abaxial surface of the bract-scale complex of specimen SX3. (D) Bract of specimen SX1 with the broken traces of the lost serrates indicated by arrowheads. Scale bars: (A, B, C), 1 cm; (D), 500 μm.
(TIF)

S2 Fig Morphology of the bract-scale complex of *Cedrus*, *Abies*, *Araucaria* and *Agathis*. (A) *Cedrus deodara*. (B) *Abies alba*. (C) *Araucaria cunninghamii*. (D) *Agathis dammara*. Scale bars: (A, B, C, D), 1 cm.
(TIF)

S3 Fig Abaxial stomatal apparatus and adaxial epidermal cells of the ovuliferous scale of extant *Pseudolarix amabilis*. (A) Inner surface of stomatal apparatus showing the monocyclic subsidiary cells. (B) Adaxial epidermal cells. Scale bars: (A), 10 μm; (B), 100 μm.
(TIF)
