# Supplementary material for: Late Miocene Pseudolarix amabilis bract-scale complex from Zhejiang, East China
Source: PLoS One. 2017 Jul 7;12(7):e0180979. doi: 10.1371/journal.pone.0180979 (PMC5501647; doi:10.1371/journal.pone.0180979)
Supplement: S1 Table — (DOCX) [file pone.0180979.s001.docx]

S1 Table. The updated fossil *Pseudolarix* *amabilis*

| Location | Longitude | Latitude | Organ | Age | References |
| --- | --- | --- | --- | --- | --- |
| Hlavačov gravel and sand, Bohemia | 13.7 °E | 50.1 °N | Cone scale | Late Oligocene/early Miocene | Bůžek and Kvaček [1]; Teodoridis [2]; Teodoridis and Sakala [3] |
| Okanagan Highlands, Southern British Columbia, Canada, and Northeastern Washington State, USA | 120.0–127.0 °W | 48.7–55.0 °N | Cone scale, pollen and seed | Eocene | Dillhoff et al. [4]; Greenwood et al. [5]; Dillhoff et al. [6]; Moss et al. [7]; Smith et al. [8]; Mathewes et al. [9]; Moss et al. [10] |
| Dembi and Amgu, Far East of Russia | 137.7 °E | 45.8 °N | Cone scale and seed | Oligocene | Akhmetiev et al. [11] |
| Qaidam Basin, China | 95.0 °E | 37.0 °N | Pollen | Pleistocene | Jiang and Robbins [12] |
| Demura, Fukui City, Japan | 136.0 °E | 36.0 °N | Seed | Early Miocene | Atsushi [13] |
| Muravyov-Amurskii Peninsula, Amur Bay, Sea of Japan | 43.3 °E | 132.0 °N | Unknown | Cretaceous | Volynets [14]; Volynets [15] |
| Enmyvaam River Basin, Central Chukotka, Northeastern Russia | 174.0 °E | 68.0 °N | Leaf | Late Cretaceous | Moiseeva and Sokolova [16] |
| Axel Heiberg Island, Canadian High Arctic | 89.0 °W | 79.9 °N | Pollen | Middle Eocene | Richter and LePage [17] |
| Meleto, Valdarno, Italy | 11.8 °E | 43.8 °N | Seed and pollen | Pliocene | Fischer and Butzmann [18] |
| Janggi Basin, Korea | 129.5 °E | 36.0 °N | Cone scale | Early Miocene | Paik et al. [19] |
| Yuyao, Zhejiang, China | 121.2 °E | 30.0 °N | Cone scale and seed | Pliocene | Li [20]; Li and Guo [21] |
| Tiantai County, Zhejiang, China | 121.2 °E | 29.2 °N | Bract-scale complex | Late Miocene | This study |

# References

1. Bůžek Č, Kvaček Z. Nové nálezy třetihorní flóry v hlavačovských štěrkopíscích u Nesuchyně na Rakovnicku. Zprávy o geol výzk v r. 1989; 1986: 22–24.
2. Teodoridis V. Tertiary flora and vegetation of the Hlavačov gravel and sand and the surroundings of Holedeč in the Most Basin (Czech Republic). Acta Mus Nat Pragae, Ser B, Hist Nat. 2002; 57: 103–140.
3. Teodoridis V, Sakala J. Early Miocene conifer macrofossils from the Most Basin (Czech Republic). N Jb Geol Paläont Abh. 2008; 250: 287–312.
4. Dillhoff R M, Leopold EB, Manchester SR. The McAbee flora of British Columbia and its relation to the early-middle Eocene Okanagan Highlands flora of the Pacific Northwest. Can J Earth Sci. 2005; 42: 151–166.
5. Greenwood DR, Archibald SB, Mathewes RW, Moss PT. Fossil biotas from the Okanagan Highlands, southern British Columbia and northeastern Washington State: climates and ecosystems across an Eocene landscape. Can J Earth Sci. 2005; 42: 167–185.
6. Dillhoff RM, Dillhoff TA, Greenwood DR, DeVore ML, Pigg KB. The Eocene Thomas Ranch flora, Allenby Formation, Princeton, British Columbia. Can Botany. 2013; 91: 514–529.
7. Moss PT, Greenwood DR, Archibald SB. Regional and local vegetation community dynamics of the Eocene Okanagan Highlands (British Columbia Washington State) from palynology. Can J Earth Sci. 2005; 42: 187–204.
8. Smith RY, Basinger JF, Greenwood DR. Early Eocene plant diversity and dynamics in the Falkland flora, Okanagan Highlands, British Columbia, Canada. Palaeobio Palaeoenv. 2012; 92: 309–328.
9. Mathewes RW, Greenwood DR, Archibald SB. Paleoenvironment of the Quilchena flora, British Columbia, during the Early Eocene Climatic Optimum 1. Can J Earth Sci. 2016: 53: 1–17.
10. Moss PT, Smith RY, Greenwood DR. A window into mid-latitudinal Early Eocene environmental variability: a high-resolution palynological analysis of the Falkland site, Okanagan Highlands, British Columbia, Canada 1. Can J Earth Sci*.* 2016; 53: 1–9.
11. Akhmetiev M, Walther H, Kvaček Z. Mid-latitude Palaeogene floras of Eurasia bound to volcanic settings and palaeoclimatic events-experience obtained from the Far East of Russia (Sikhote-Alin’) and Central Europe (Bohemian Massif). Acta Mus Nat Pragae, Ser B, Hist Nat. 2009; 65: 61–129.
12. Jiang DX, Robbins EI. Quaternary palynofloras and paleoclimate of the Qaidam Basin, Qinghai Province, northwestern China. Palynology 2000; 24: 95–112.
13. Atsushi Y. Plant megafossil assemblage from the Lower Miocene Ito-o Formation, Fukui Prefecture, Central Japan. Mem Fukui Prefect Dinosaur Mus. 2008: 7: 1–24.
14. Volynets E. The Aptian–Cenomanian flora of Primor’e, Part 1: floral assemblages. Stratigr. Geol Correl. 2005; 13: 613–631.
15. Volynets E. Cretaceous deposits and flora of the Muravyov-Amurskii Peninsula (Amur Bay, Sea of Japan). Stratigr Geol Correl. 2015; 23: 281–299.
16. Moiseeva M, Sokolova A. New data on the composition and age of the Ust’-Emuneret flora from the Enmyvaam River basin (Central Chukotka). Stratigr Geol Correl. 2014; 22: 269–286.
17. Richter SL, LePage BA. A high-resolution palynological analysis, Axel Heiberg Island, Canadian High Arctic. In: LePage BA, Williams CJ, Yang H, editors. The Geobiology and Ecology of *Metasequoia*. Berlin: Springer; 2005. pp. 137–158.
18. Fischer TC, Butzmann R. The Pliocene macro-and microflora of lacustrine sediments from Meleto (Valdarno, N. Italy) and its ecological, palaeobiogeographical and climatic interpretation. Cainozoic Res. 2006; 5: 71–88.
19. Paik IS, Kim HJ, Kim K, Jeong EK, Kang HC, Leea HI, et al. Leaf beds in the Early Miocene lacustrine deposits of the Geumgwangdong Formation, Korea: Occurrence, plant-insect interaction records, taphonomy and palaeoenvironmental implications. Rev Palaeobot Palyno. 2012; 170: 1–14.
20. Li HM. Neogene floras from eastern Zhejiang, China. In: Whyte RO, editor. The evolution of the East Asian environment Vol II palaeobotany, palaeozoology and palaeoanthropology. Hong Kong: University of Hong Kong; 1984. pp. 461–466.
21. Li HM, Guo SX. Spermatophyte. In: Nanjing Institute of Geology and Mineral Resources, editor. Atlas of paleontology in East China (III) Mesozoic and Cenozoic. Beijing: Geology Publishing House; 1982. pp. 280–316.
