# Supplementary material for: Late Miocene Pseudolarix amabilis bract-scale complex from Zhejiang, East China
Source: PLoS One. 2017 Jul 7;12(7):e0180979. doi: 10.1371/journal.pone.0180979 (PMC5501647; doi:10.1371/journal.pone.0180979)
Supplement: S2 Table — (DOCX) [file pone.0180979.s002.docx]

S2 Table. The occurrence of living *Pseudolarix amabilis* in China

| Locations | Longitude (°E) | Latitude (°N) |
| --- | --- | --- |
| Wanzhou District, Sichuan Province | 108.41 | 30.81 |
| Lichuan City, Hubei Province | 108.94 | 30.29 |
| Enshi City, Hubei Province | 109.49 | 30.27 |
| Xing'an County, [Guangxi](javascript:void(0);) [Zhuang](javascript:void(0);) [Autonomous](javascript:void(0);) [Region](javascript:void(0);) | 110.59 | 25.60 |
| Nan County, Hunan Province | 112.41 | 29.37 |
| Nanyue District, Hunan Province | 112.73 | 27.24 |
| Nanyue District, Hunan Province | 112.74 | 27.23 |
| Hengshan County, Hunan Province | 112.81 | 27.22 |
| Shen County, Hubei Province | 113.31 | 31.86 |
| Tongchen County, Hubei Province | 113.82 | 29.25 |
| Chongyang County, Hubei Province | 114.04 | 29.56 |
| Wuhan City, Hubei Province | 114.31 | 30.59 |
| Tonggu County, Jiangxi Province | 114.37 | 28.52 |
| Xiushui County, Jiangxi Province | 114.41 | 29.01 |
| Xin County, Henan Province | 114.88 | 31.64 |
| Yingshan County, Hubei Province | 115.68 | 30.74 |
| Lushan, Jiangxi Province | 115.99 | 29.67 |
| Jiujiang City, Jiangxi Province | 116.00 | 29.71 |
| Hengshan City, Hunan Province | 116.32 | 31.40 |
| Shucheng County, Anhui Province | 116.95 | 31.46 |
| Jiangle county, Fujian Province | 117.47 | 26.73 |
| Qingyang county, Anhui Province | 117.85 | 30.64 |
| Chongan County, Fujian Province | 118.04 | 27.76 |
| Xiamen City, Fujiang Province | 118.09 | 24.88 |
| Pucheng County, Fujian Province | 118.54 | 27.92 |
| Nanjing City, Jiangshu Province | 118.80 | 32.06 |
| Suichang County, Zhejiang Province | 119.28 | 28.59 |
| Linan City, Zhejiang Province | 119.37 | 30.20 |
| Liyang County, Jiangshu Province | 119.38 | 31.42 |
| Guangde County, Anhui Province | 119.42 | 30.89 |
| Tonglu County, Zhejiang Province | 119.69 | 29.79 |
| Yingxing County, Jiangshu Province | 119.82 | 31.38 |
| Fuyang District, Zhejiang Province | 119.96 | 30.05 |
| Hangzhou City, Zhejiang Province | 120.17 | 30.27 |
| Qingdao City, Shandong Province | 120.38 | 36.07 |
| Tiantai County, Zhejiang Province | 120.98 | 29.14 |
| Leqing County, Zhejiang Province | 121.02 | 28.25 |
| Taiyuan City, Shanxi Province | 122.55 | 37.87 |
