# Supplementary figures and images for: Late Miocene Pseudolarix amabilis bract-scale complex from Zhejiang, East China
Source: PLoS One. 2017 Jul 7;12(7):e0180979. doi: 10.1371/journal.pone.0180979 (PMC5501647; doi:10.1371/journal.pone.0180979)

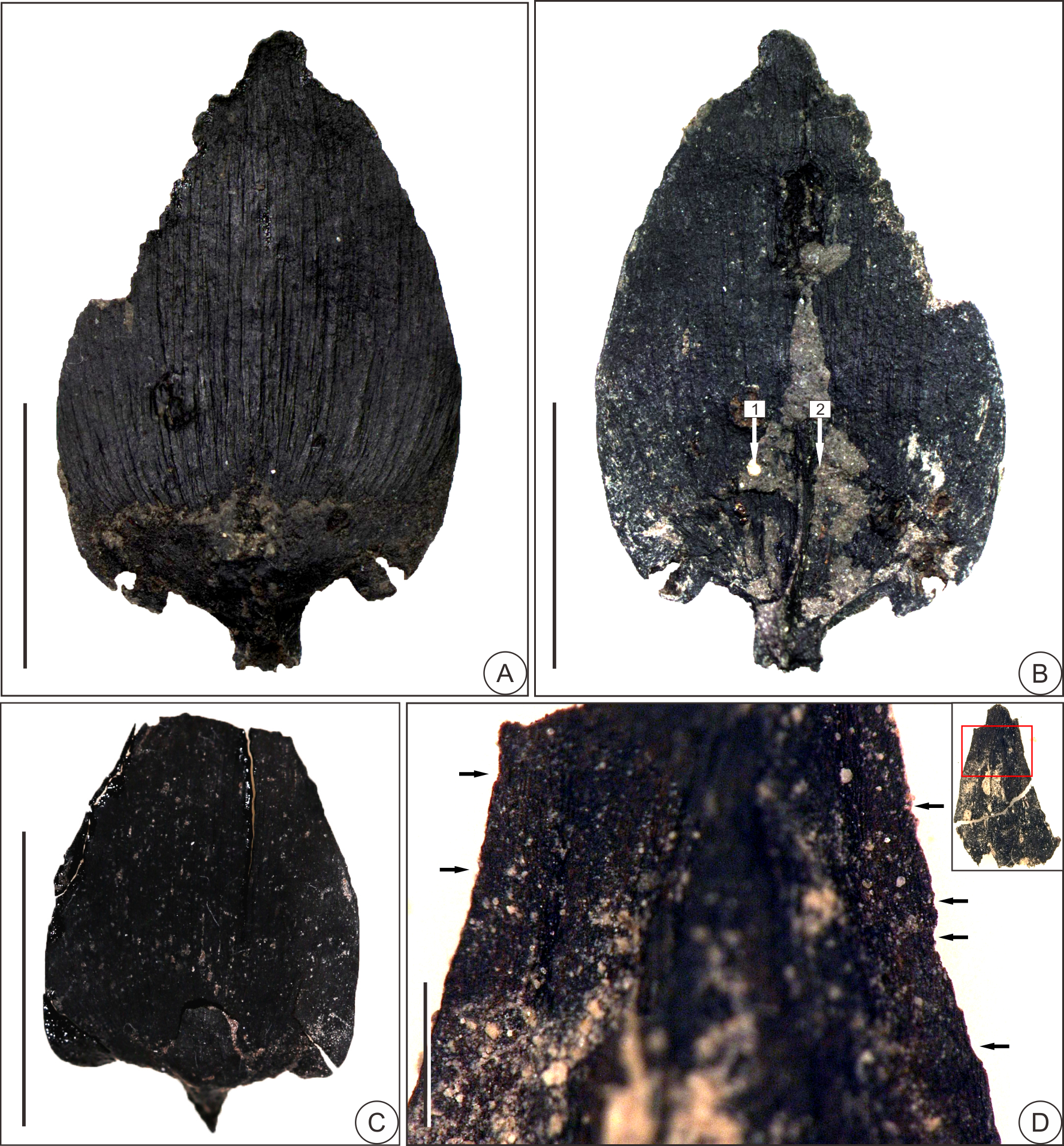

Supplement: S1 Fig — (A, B) Ovuliferous scale of specimen SX2. (A) Abaxial surface. (B) Adaxial surface with arrowheads 1 and 2 pointing to the two seed impressions. (C) Abaxial surface of the bract-scale complex of specimen SX3. (D) Bract of specimen SX1 with the broken traces of the lost serrates indicated by arrowheads. Scale bars: (A, B, C), 1 cm; (D), 500 μm. (TIF) [file pone.0180979.s003.tif]

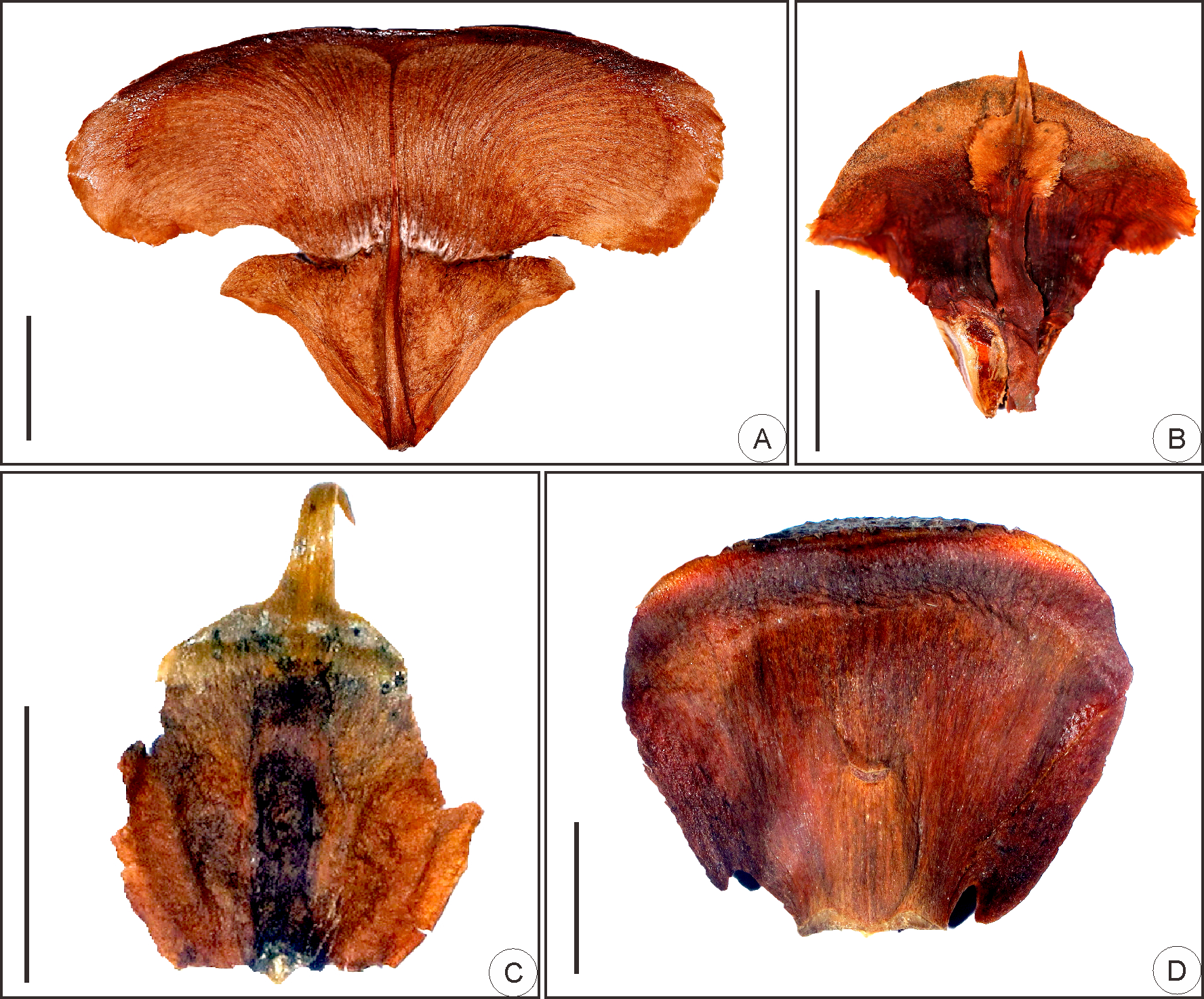

Supplement: S2 Fig — (A) Cedrus deodara. (B) Abies alba. (C) Araucaria cunninghamii. (D) Agathis dammara. Scale bars: (A, B, C, D), 1 cm. (TIF) [file pone.0180979.s004.tif]

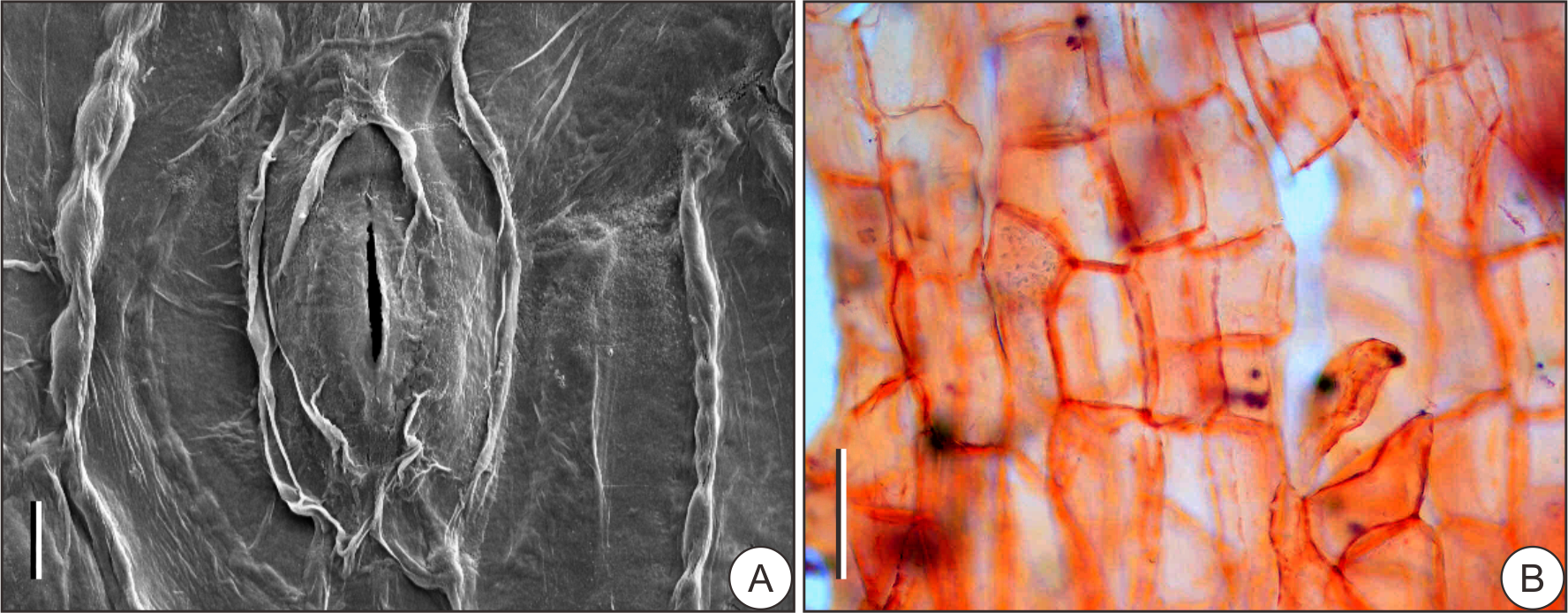

Supplement: S3 Fig — (A) Inner surface of stomatal apparatus showing the monocyclic subsidiary cells. (B) Adaxial epidermal cells. Scale bars: (A), 10 μm; (B), 100 μm. (TIF) [file pone.0180979.s005.tif]
